# Combined RhoA morpholino and ChABC treatment protects identified lamprey neurons from retrograde apoptosis after spinal cord injury

**DOI:** 10.3389/fncel.2023.1292012

**Published:** 2023-12-21

**Authors:** Jianli Hu, Guixin Zhang, William Rodemer, Li-Qing Jin, Michael E. Selzer

**Affiliations:** ^1^Center for Neural Repair and Rehabilitation (Shriners Hospitals Pediatric Research Center), Lewis Katz School of Medicine (LKSOM) at Temple University, Philadelphia, PA, United States; ^2^Department of Neural Sciences, LKSOM, Philadelphia, PA, United States; ^3^Center for Neurodegenerative Disease Research, Perelman School of Medicine, University of Pennsylvania, Philadelphia, PA, United States; ^4^Department of Neurology, LKSOM, Philadelphia, PA, United States

**Keywords:** neuronal apoptosis, axon regeneration, caspases, ChABC, RhoA, Akt, SCI, lamprey

## Abstract

Previously, we reported that RhoA knockdown by morpholino antisense oligonucleotides (MOs), and enzymatic digestion of chondroitin sulfate proteoglycans (CSPGs) at the site of injury with chondroitinase ABC (ChABC), each can reduce retrograde neuronal apoptosis after spinal cord transection in the lamprey. To elucidate the mechanisms in neuronal survival and axon regeneration, we have investigated whether these two effects are additive *in vivo*. We used lampreys as a spinal cord injury model. MOs were used to knockdown RhoA and Chondroitinase ABC (ChABC) was used to digest CSPGs *in vivo*. Retrograde labeling, fluorochrome-labeled inhibitor of caspase activity (FLICA), immunohistochemistry, and western blots were performed to assess axonal regeneration, neuronal apoptotic signaling and Akt activation. Four treatment combinations were evaluated at 2-, 4-, and 10-weeks post-transection: (1) Control MO plus enzyme buffer (Ctrl); (2) control MO plus ChABC; (3) RhoA MO plus enzyme buffer (RhoA MO); and (4) RhoA MO plus ChABC (RhoA MO + ChABC). Consistent with our previous findings, at 4-weeks post-transection, there was less caspase activation in the ChABC and RhoA MO groups than in the Ctrl group. Moreover, the RhoA MO plus ChABC group had the best protective effect on identified reticulospinal (RS) neurons among the four treatment combinations. At 2 weeks post-transection, when axons have retracted maximally in the rostral stump and are beginning to regenerate back toward the lesion, the axon tips in the three treatment groups each were closer to the transection than those in the Ctr MO plus enzyme buffer group. Long-term axon regeneration also was evaluated for the large, individually identified RS neurons at 10 weeks post-transection by retrograde labeling. The percent regenerated axons in the RhoA MO plus ChABC group was greater than that in any of the other groups. Akt phosphorylation levels at threonine 308 was quantified in the identified RS neurons by western blots and immunofluorescence. The RhoA MO plus ChABC treatment enhanced pAkt-308 phosphorylation more than any of the other treatment groups. Although some of the effects of CSPGs are mediated through RhoA activation, some growth-inhibiting mechanisms of RhoA and CSPGs are independent of each other, so combinatorial therapies may be warranted.

## Introduction

Spinal cord injury (SCI) can cause devastating permanent paralysis for which there is still no effective biological treatment. The bacterial enzyme chondroitinase ABC (ChABC) has been widely studied as a treatment to promote axon sprouting and functional recovery after SCI (Bradbury et al., 2002; Cafferty et al., 2007, 2008; Starkey et al., 2012; Hu et al., 2021). This enzyme can digest the sugar chains of the chondroitin sulfate proteoglycans (CSPGs), which was at first considered as a physical barrier to axon re-growth after injury, but later identified as a substrate interacting with specific receptors (Shen et al., 2009; Fisher et al., 2011; Sharma et al., 2012). Removal of the sugar chains of CSPGs has been reported to promote axon regeneration and functional recovery after SCI in many different animal models (Bradbury et al., 2002; Cafferty et al., 2007, 2008; Starkey et al., 2012; Hu et al., 2021). In the past decade or so, the receptor-like protein tyrosine phosphatases (RPTPs), protein tyrosine phosphatase sigma (PTPσ) and leukocyte common antigen-related phosphatase (LAR), have been identified as transmembrane receptors for CSPGs (Shen et al., 2009; Fisher et al., 2011; Sharma et al., 2012). Downregulation or inhibition of these CSPG receptors also has been shown to enhance axon growth after SCI (Shen et al., 2009; Fisher et al., 2011; Lang et al., 2015; Xu et al., 2015), which further indicates the importance of CSPGs and indirectly suggests that ChABC might be a critical tool in the treatment of SCI. At least part of the effect of the RPTPs is to activate RhoA (Fisher et al., 2011), and inhibitors of RhoA also can enhance axon growth after SCI (Hu et al., 2017). If all the effects of CSPGs work through activation of RPTPs, signaling their inhibitory effects by activating RhoA, then the effects of RhoA knockdown should not be further enhanced by ChABC treatment. Evaluation of the signaling pathways downstream of the CSPG receptors, and their relationship to axon regeneration, are difficult using *in vivo* mammalian models of SCI, which, because of the very high barrier to axon regeneration posed by the dense glial and fibrotic scar, mostly involve incomplete lesions. Combined with the anatomical complexity and opacity of the mammalian CNS, the presence of spared axons makes it difficult to distinguish true regeneration from collateral sprouting. For this reason, we have used complete spinal cord transection in the lamprey, where axon regeneration and functional recovery does occur spontaneously, though incompletely, and where axon regeneration can be evaluated in the living animal.

The brainstem of the lamprey has individually identified reticulospinal (RS) neurons, which have defined perikaryal survival and axon regeneration probabilities after SCI (Davis and McClellan, 1994; Jacobs et al., 1997). We took advantages of these identified neurons and found that both morpholino (MO)-induced RhoA knockdown and ChABC treatment *in vivo* each individually reduce retrograde neuronal apoptotic signaling after spinal cord transection (Hu et al., 2017, 2021). Both MO knockdown of RhoA and treatment with ChABC *in vivo* each promoted axon regeneration and/or reduced axon retraction early after transection, and either treatment also had beneficial long-term effects on axon regeneration (Hu et al., 2017, 2021).

Previous research from other labs showed that PTEN knockout leads to potent CNS axon growth after injury (Park et al., 2008; Liu et al., 2010; Du et al., 2015), and the signaling molecules downstream of PTEN that mediate this effect, including Akt, have been studied extensively (Liu et al., 2011). Akt activation promoted axon regeneration in the optic nerve and survival of retinal ganglion cells (RGC) (Yang et al., 2014; Guo et al., 2016). Similarly, in the lamprey, ChABC-enhanced axonal regeneration at 10 weeks after SCI was accompanied by widespread enhancement of Akt activation (pAkt-308) in the brain, and protection of RS neurons from retrograde apoptosis (Hu et al., 2021).

Taking all of this into account, the present study aimed to determine whether after SCI in the lamprey, simultaneous use of ChABC and MO-induced RhoA knockdown provide more benefit in promoting axon regeneration and inhibiting apoptotic signaling than either treatment alone.

## Materials and methods

### Spinal cord transection and combinatorial treatments

Wild-type larval lampreys, Petromyzon marinus, 10–14 cm in length (4–5 years old), were obtained from streams of Lake Michigan and maintained in freshwater tanks at room temperature (RT). For spinal cord transection (TX), animals were anesthetized by immersion in 0.1% tricaine methanesulfonate, and the spinal cord was exposed by an incision along the dorsal midline centered at the level of the fifth gill. TX of the spinal cord was performed with Castroviejo scissors. Completeness of TX was confirmed by retraction and visual inspection of the cut ends.

For combinatorial treatments, a pledget of Gelfoam soaked with 1 μl Ctr MO or RhoA MO was inserted into the TX site under a dissecting microscope. TXed lampreys recovered on ice for 2 h, the Gelfoam was taken out gently and the animals were returned to fresh-water tanks at RT for 1 week. Then the lampreys were re-anesthetized and the spinal cord was re-exposed along the original dorsal midline incision. A pledget of Gelfoam soaked either with ChABC (Cat# C2905, Sigma-Aldrich) dissolved in 1 μl enzyme buffer or with 1 μl enzyme buffer alone was inserted into the TX site, and a similar pledget was placed gently on the dorsal surface of the spinal cord spanning the injury site. TXed lampreys recovered on ice for 2 h and then were returned to freshwater tanks at RT for another 1, 3, or 10 weeks, at which times axon regeneration was assessed, or the animals were studied by fluorochrome-labeled inhibitor of caspase activity (FLICA) assay, or by immunofluorescence for Akt phosphorylation at threonine 308 (pAkt-308) (Table 1).

**TABLE 1:** Number of lampreys used in each set of experiments.

Groups	2 weeks FLICA	4 weeks FLICA	2 weeks axon regeneration	10 weeks axon regeneration	pAkt-308 western blots	pAkt-308 immunofluorescence
**Number of lampreys used in each set of experiments**						
CtrMO + Buffer	5	5	4	5	4	11
CtrMO + ChABC	5	5	5	5	4	12
RhoA MO + Buffer	5	5	4	5	4	9
RhoA MO + ChABC	5	5	4	5	4	14

### Axon regeneration

To assess the effects of combinatorial treatments on axon regeneration at 2 weeks after TX (Table 1), lampreys were anesthetized, and lengths of spinal cord spanning the TX site were removed, pinned carefully onto Sylgard strips, and fixed with 4% paraformaldehyde (PFA)/PBS for 2 h at RT. The spinal cords were washed with PBS 30 min/time for 3 times. All of the axon tips were imaged with fluorescence microscopy under the same parameters. To assess long-term axon regeneration, 10 weeks after the first TX at the level of the 5th gill, a second TX was performed 5 mm caudal to the first TX, and a pledget of Gelfoam soaked in 5% dextran tetramethylrhodamine (DTMR; Cat# D1817, Thermo Fisher Scientific) was inserted into the 2nd TX gap. The lampreys recovered on ice for 2 h and then were returned to freshwater tanks at RT for another 1 week to allow DTMR to label the regenerated axon segments (Table 1). The lampreys were anesthetized, and the brains were dissected out carefully, pinned onto the Sylgard, and fixed with 4% PFA/PBS for 2 h at RT. The brains were washed 3 times with PBS, 30 min/wash. All brains were imaged by fluorescence microscopy using the same parameters.

### FLICA on whole-mounted lamprey brains

After the recovery times described above, animals were re-anesthetized by immersion in saturated benzocaine solution. Brains were dissected out in ice-cold lamprey Ringer (110 mM NaCl, 2.1 mM KCl, 2.6 mM CaCl_2_, 1.8 mM MgCl_2_, and 10 mM Tris buffer; pH 7.4). The posterior and cerebrotectal commissures of the freshly dissected brains were split along the dorsal midline. Brains were incubated immediately at 4°C for 1 h in 150 μL 1 × FLICA labeling solution (Image-iT™ Live Green Poly-Caspases Detection Kit, Cat# I35104, Molecular Probes; or Green Caspase-3 Staining Kit, Cat# PK-CA577-K183-25, PromoKine), which was diluted with phosphate buffered saline (PBS). Afterward, brains were washed five times with 1X wash buffer on a rotator at 4°C, 5 min per time. The alar plates of brains were deflected laterally and pinned flat to a small strip of Sylgard (Dow Corning Co., USA). The tissue was fixed in 4% PFA in PBS for 2 h at RT, and then washed 3 times in PBS at RT. Fluorescence images of brains were captured immediately with a Nikon 80i microscope. The whole procedure was conducted in the dark and all the samples were carefully protected from light. The brains were placed in 70% EtOH and kept at −20°C for PTPσ mRNA *in situ* hybridization (ISH). Control experiments were performed using brains from lampreys without spinal cord TX (Table 1). All images were acquired using the same parameters.

### Calculation of probabilities of FLICA positivity for identified RS neurons

The number of FLICA-positive neurons were counted separately for each of the individually identified RS neurons in each brain. Then, for each of the individually identified RS neurons, the number of FLICA-positive neurons was divided by the total number of neurons (2) of that individual type (FLICA-positive and -negative) in each brain, and the percentages were considered the probability of FLICA positivity for each of the identified RS neurons. For example, if among the five brains treated with RhoA MO plus ChABC at 2 weeks post-TX, four I1 neurons were FLICA-positive. Since each brain has two I1 neurons, the total numbers of I1 neurons are 2 × 5 = 10. Thus, the probability of FLICA positive is (4÷10) × 100% = 40%. Previously, the 18 pairs of individually identified RS neurons have been individually characterized regarding the probability of whether they will regenerate by 10 weeks post-transection (Jacobs et al., 1997). Thus, we performed a correlation analysis and simple linear regression analysis between FLICA-positive probabilities and regeneration probabilities for all the individually identified RS neurons in each of the four combinatorial treatment groups.

### Western blotting

Lamprey brains were dissected out from the olfactory lobe to the obex to investigate the expression of Akt (Table 1). The tissues were snap-frozen in liquid nitrogen and homogenized in cold lysis buffer (Cat# C3228, Sigma-Aldrich) supplemented with 1 × protease inhibitor cocktail (Cat# P8341, Sigma-Aldrich). After brief centrifugation to remove debris, the total protein concentration in supernatants was determined using Bio-Rad (Hercules, CA, USA) DC protein assay reagents (Cat# 500-0006, Bio-Rad). After 10 min of heating at 75°C in loading buffer (Cat# NP 0007, Invitrogen) supplemented with reducing reagent (Cat# NP 0004, Invitrogen), 25 μg of protein were loaded from each sample. The protein was separated in 4–12% NuPAGE^®^ Bis-Tris gradient mini gels (Cat# NP 0321BOX, Invitrogen), and transferred onto a PVDF membrane, using a Bio-Rad trans blot apparatus. The membranes were blocked in 5% non-fat dry milk in TRIS-buffered saline (TBS) for 1 h at RT. Membranes were probed with an anti-pAkt-308 antibody (Cat# 2965, Cell Signaling) diluted 1:1000 at 4°C overnight, anti-Akt-pan antibody (Cat# 4691, Cell Signaling) diluted 1:4000 at RT for 1 h, anti-RhoA antibody (Cat# SAB 1400017, Sigma) diluted 1:600 at 4°C overnight, or anti-Actin (Cat# MAB1501, Chemicon) diluted 1:10,000 at 4°C overnight. After washes with TBS, the blots were incubated with secondary antibodies IRDye 800CW goat anti-rabbit IgG (Cat# 926-32211, LI-COR), IRDye 680RD goat anti-rabbit IgG (Cat# 926-68071, LI-COR), IRDye 800CW goat anti-mouse IgG (Cat# 926-32210, LI-COR), or IRDye 680RD goat anti-mouse IgG (Cat# 926-68070, LI-COR) or at 1: 20,000 for 1 h at RT in the dark. The blots were washed 3 times with TBS, 10 min each, scanned and quantified with an Odyssey CLx (LI-COR), and processed with Adobe Photoshop (San Jose, CA, USA).

### Immunohistochemistry

We routinely test every antibody for sensitivity and specificity before using it in our lab. This includes demonstration of correct bands on western blotting and negative control staining on immunohistochemistry. For the primary antibody pAkt-308 and secondary antibody donkey anti-rabbit Alexa Fluor^®^ 594 used in the immunohistochemical staining, this testing showed no non-specific labeling. To measure levels of pAkt-308 in individual identified neurons, (Table 1) after fixation, dehydration and paraffin embedding, 10 μm thick paraffin serial sections were mounted onto glass slides, de-paraffinized, rehydrated, and washed in PBS. Antigen retrieval was performed as follows: Sections were immersed in the sodium citrate buffer (10 mM sodium citrate, pH 6.0). The buffer was boiled for 20 min, and the sections allowed to cool for 20 min at RT. Sections were rinsed in PBS twice, 5 min each time. All the sections were blocked with 10% FBS/0.2% Tween-20/PBS for 1 h at RT, and incubated overnight with primary antibody anti-pAkt-308 (Cat# 2965, Cell Signaling) diluted 1:1000 in 10% FBS/0.2% Tween-20/PBS at 4°C. Sections were washed 3 times with PBS, 10 min each and then incubated with donkey anti-rabbit Alexa Fluor^®^ 594 (Cat# A21207, Thermo Fisher Scientific) diluted 1:200 in blocking buffer for 1 h at RT. All sections were washed 3 times with PBS, 10 min each time. After incubation in the secondary antibody, sections were washed 3 times with PBS, 10 min each time. Autofluorescence was carefully quenched with the TrueView kit (Cat# SP-8400, Vector TrueView). Sections were mounted with Fluoromount-G (Cat# 0100-01, Southern Biotech). DAF-488 and pAkt-308 fluorescence signaling were captured with a Nikon 80i microscope under consistent parameters to allow quantification of pAkt-308 fluorescence. All brain sections with identified neurons were collected and quantified with NIS-Elements AR 3.10. For each brain section, all the identified neurons that were filled with MOs were outlined and pAkt-308 intensity was measured. Background fluorescence intensity was measured by outlining the area adjacent to the brain. The fluorescence intensity for each section was calculated as follows: the background fluorescence intensity was subtracted from the mean fluorescence intensity within identified neurons in the same section. For each animal, the average fluorescence intensity from all the sections was calculated. Then an overall mean fluorescence was calculated as the mean of all these average intensities.

### Statistical analysis

Data sets were analyzed with GraphPad Prism 10.0.3 for Windows, GraphPad Software, Boston, MA, USA, www.graphpad.com. Normally distributed data were analyzed by InStat. The effects of combinatorial treatments on apoptosis signaling were determined for individual identified neurons, by comparing FLICA labeling for the same RS neurons in different treatment groups. To investigate whether the intensity of retrograde apoptotic signaling is correlated with the regenerative probabilities in each individually identified RS neuron, we performed a Pearson correlation analysis and a simple linear regression analysis in each of the four combinatorial treatment groups at 2 and 4 weeks post-transection. The effects of combinatorial treatments on early stage axon regeneration were determined by comparing the distances of the axon tips from the TX site. The effects of combinatorial treatments on long-term axon regeneration were determined by comparing the numbers of brain neurons retrogradely labeled with DTMR. For Western blots, to avoid between-blot variation, all the groups were normalized against actin loading controls. Then the experimental groups were compared with their respective normalized control groups, whose relative densities were assigned a value of 1. The effects of combinatorial treatments on pAkt-308 were determined by measuring the pAkt-308 immunofluorescence intensity. All data underwent one-way Analysis of Variance (ANOVA) followed by the Tukey’s multiple comparisons test. All values were expressed as mean ± SEM.

## Results

### The efficiency of ChABC treatment and RhoA knockdown

Previously we have used ChABC application in lamprey spinal cord and have validated their effects over CSPG digestion *in vivo* (Hu et al., 2021). The stumps of CSPGs digested by ChABC were labeled by 2B6 immunostaining at 1 day, 1 and 2 weeks after transection in sagittal sections of spinal cord (Supplementary Figures 1A–C). The use of RhoA MO *in vivo* can efficiently knockdown RhoA expression in brain and spinal cord (Hu et al., 2017). Transverse sections of spinal cord showed dramatic decreases in RhoA expression levels in axons treated with RhoA MO, compared to the ones treated with Ctr MO (Supplementary Figures 2C, D). Neurofilament staining highlighted the axons that were treated with either Ctr MO or RhoA MO similarly (Supplementary Figures 2A, B), which suggests the integrity of axoplasm was well-preserved and the quantities of neurofilaments were similar between these two groups. Thus in Supplementary Figure 2D, the reduced level of RhoA in the axons was not due to protein leakage or loss by axon damage but was indeed due to the *in vivo* RhoA MO knockdown. We also performed western blots on brain and spinal cord protein extracts to test the efficiency of RhoA knockdown and showed obviously lower RhoA protein levels after RhoA MO application in both brain and spinal cord (Supplementary Figure 4).

### Effects combinatorial treatments on retrograde neuronal apoptosis signaling after spinal cord TX

**FIGURE 1:**
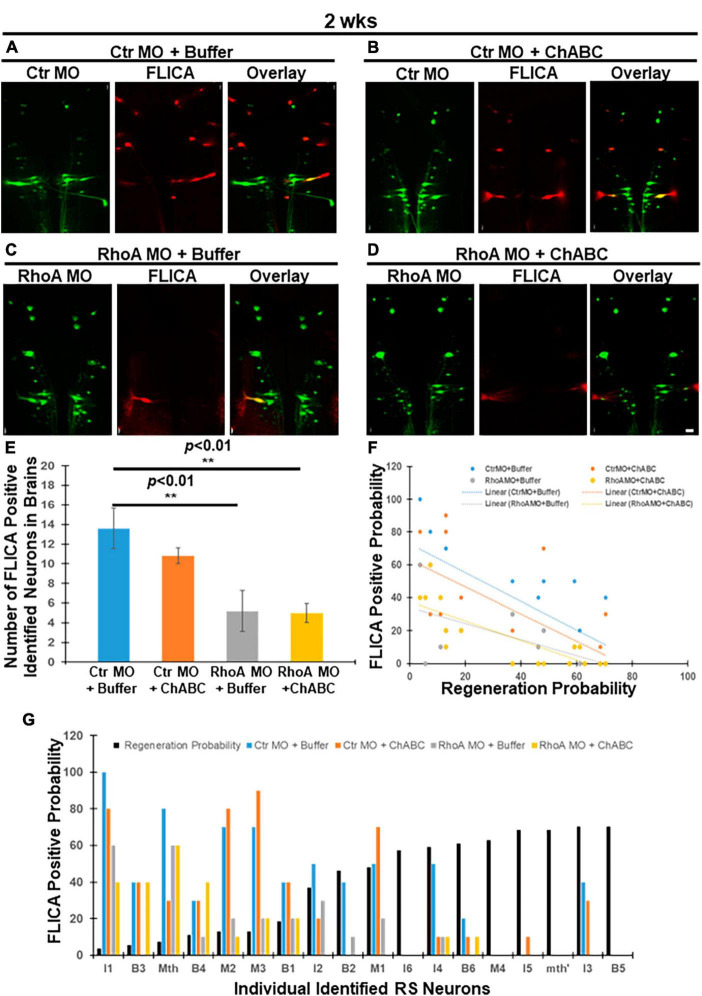
Effects of combinatorial treatments on retrograde apoptosis signaling at 2 weeks post-TX. FLICA was used to detect neurons undergoing apoptosis after TX. Lampreys were treated with one of four combinatorial therapies and examined at 2 weeks post-TX as described in section “Materials and methods”: Ctr MO (applied at time of TX) plus enzyme buffer (applied at 1 week post-TX), Ctr MO (applied at time of TX) plus ChABC (in enzyme buffer, applied at 1 week post-TX), RhoA MO (applied at time of TX) plus enzyme buffer (applied at 1 week post-TX), and RhoA MO (applied at time of TX) plus ChABC (in enzyme buffer, applied at 1 week post-TX). **(A)** FLICA-positive RS neurons (red) in the brain that were retrogradely labeled with Ctr MO (green) and treated with enzyme buffer. **(B)** FLICA-positive RS neurons (red) that were retrogradely labeled with Ctr MO (green) and treated with ChABC. **(C)** FLICA-positive RS neurons (red), retrogradely labeled with RhoA MO (green) and treated with enzyme buffer. **(D)** FLICA-positive RS neurons (red) which were retrogradely labeled with RhoA MO (green) and treated with ChABC. **(E)** Graph shows the number of FLICA positive identified RS neurons in lamprey brains after different combinatorial treatments. **(F)** Graph shows the probabilities for each of the identified RS neurons to be FLICA positive at 2 weeks post-TX, after different combinatorial treatments. **(G)** Negative correlation between the FLICA-positive probabilities of each individual RS neuron after different combinatorial treatments. The neurons are arrayed according to their previously determined regeneration probabilities (black bars) under control conditions, measured at 12 weeks post-TX (Jacobs et al., 1997). **p < 0.01, *n* = 5 lampreys per group. Error bars: SEM. Scale bar: 100 μm.

Fluorochrome-labeled inhibitor of caspase activity has been used to detect neurons undergoing apoptosis after spinal cord TX in lampreys (Barreiro-Iglesias and Shifman, 2012; Hu et al., 2013, 2017, 2021). As we reported previously, either RhoA MO knockdown or ChABC treatment *in vivo* can reduce this retrograde neuronal apoptotic signaling (Hu et al., 2017, 2021). In the current study, lampreys with spinal cord TX were treated with four different combinatorial therapies: Ctr MO plus enzyme buffer, Ctr MO plus ChABC dissolved in enzyme buffer, RhoA MO plus enzyme buffer, and RhoA MO plus ChABC dissolved in enzyme buffer. Ctr MO with ChABC treatment did not significantly affect the number of polycaspase-positive (FLICA +) RS neurons at 2 weeks post-TX compared with Ctr MO plus enzyme buffer (Figures 1A vs. B, E; 13.6 ± 2.09 vs. 10.8 ± 0.80). RhoA MO and enzyme buffer significantly reduced the number of polycaspase-positive RS neurons compared with Ctr MO plus enzyme buffer (controls) at 2 weeks post-TX (Figures 1A vs. C, E; 13.6 ± 2.09 vs. 5.2 ± 2.06; *p* < 0.01), similarly to our previous findings with RhoA MO treatment only (Hu et al., 2017). There was no difference between RhoA MO plus enzyme buffer and RhoA MO plus ChABC treatment (Figures 1C vs. D, E; 5.2 ± 2.06 vs. 5.0 ± 0.95). Because the MO was applied at the time of TX, it had been present for 2 weeks, whereas enzyme buffer with or without ChABC was applied at 1 week post TX, and therefore was present for only 1 week. Not surprisingly, RhoA MO plus ChABC greatly reduced the number of polycaspase-positive RS neurons compared with Ctr MO plus enzyme buffer (controls) at 2 weeks post-TX (Figures 1A vs. D, E; 13.6 ± 2.09 vs. 5.0 ± 0.95, *p* < 0.01). Moreover, the intensity of retrograde apoptotic signaling was inversely correlated with the regenerative probabilities in each individually identified RS neurons in all the four different combinatorial treatment groups (Figures 1F, G; Ctr MO + Buffer: *r* = −0.7493, *p* < 0.001; Ctr MO plus ChABC: *r* = −0.6966, *p* < 0.01; RhoA MO plus Buffer: *r* = −0.6636, *p* < 0.01; RhoA MO + ChABC: *r* = −0.8158, *p* < 0.0001). The linear regression equation for Ctr MO treatment only was *Y* = −0.8736 X + 72.84 (*p* < 0.001); for Ctr MO plus ChABC treatment, it was *Y* = −0.8248 X + 63.10 (*p* < 0.01); for RhoA MO treatment only, it was *Y* = −0.4878 X + 34.02 (*p* < 0.01); for RhoA MO plus ChABC treatment, it was *Y* = −0.5892 X + 37.53 (*p* < 0.0001) (Figure 1F).

**FIGURE 2:**
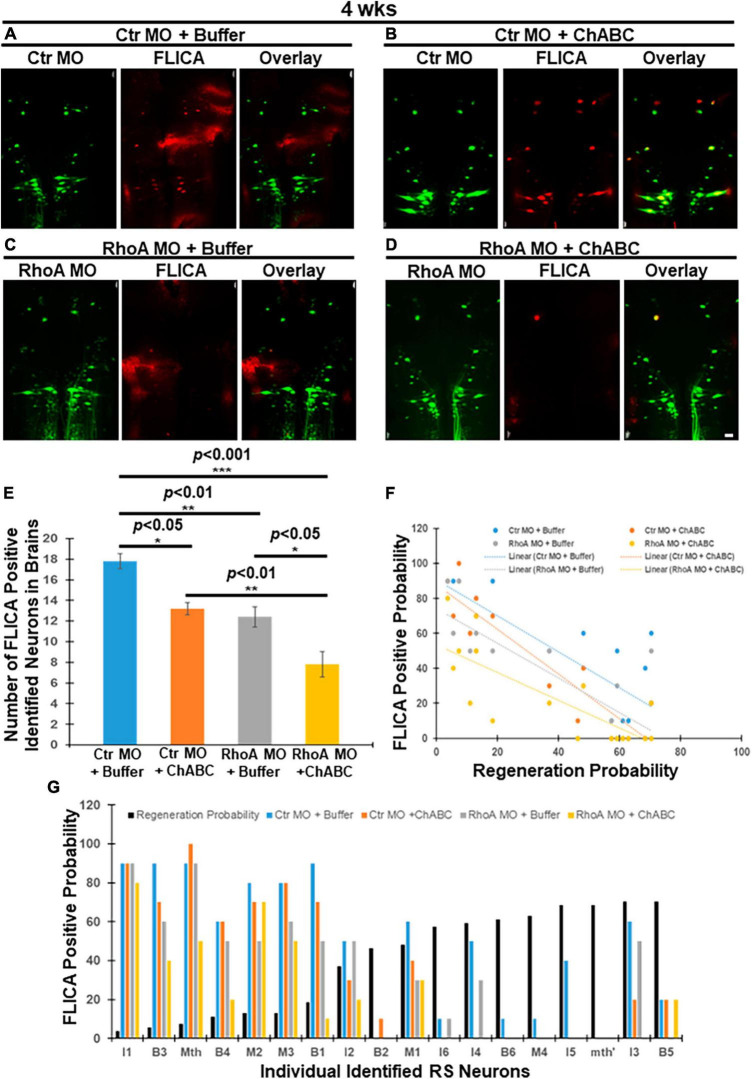
Effects of combinatorial treatments on retrograde apoptosis signaling at 4 weeks post-TX. Lampreys were treated as in Figure 1, but examined at 4 weeks post-TX. **(A)** FLICA positive RS neurons (red) in the brain were retrogradely labeled with Ctr MO (green) plus enzyme buffer. **(B)** FLICA-positive RS neurons (red) retrogradely labeled with Ctr MO (green) and treated with ChABC. **(C)** FLICA positive identified neurons (red) retrogradely labeled with RhoA MO (green) and treated with enzyme buffer. **(D)** FLICA positive identified neurons (red) which retrogradely labeled with RhoA MO (green) and treated with ChABC (dissolved with Enzyme buffer). **(E)** Graph shows the number of FLICA-positive identified RS neurons in lamprey brains after different combinatorial treatments. **(F)** The probabilities that each of the identified RS neurons are FLICA positive after different combinatorial treatments at 4 weeks transection. **(G)** Negative correlation among identified RS neurons between the probability that it is FLICA positive after different combinatorial treatments, and its previously determined regeneration probability. *p < 0.05, **p < 0.01, ***p < 0.001, *n* = 5 lampreys per group. Error bars: SEM. Scale bar: 100 μm.

At 4 weeks post-TX, groups treated with Ctr MO plus ChABC, RhoA MO plus enzyme buffer, and RhoA MO plus ChABC, all had greatly reduced numbers of polycaspase-positive (FLICA +) RS neurons, compared with animals treated with Ctr MO plus enzyme buffer (Figures 2A vs. B, 17.8 ± 0.73 vs. 13.2 ± 0.58, *p* < 0.05; Figures 2A vs. C, 17.8 ± 0.73 vs. 12.4 ± 0.98, *p* < 0.01; Figures 2A vs. D, 17.8 ± 0.73 vs. 7.8 ± 1.24, *p* < 0.001; Figure 2E). Moreover, addition of ChABC treatment to RhoA MO significantly further reduced the number of apoptotic neurons compared with animals receiving only RhoA MO plus enzyme buffer (Figures 2C vs. D, E; 12.4 ± 0.98 vs. 7.8 ± 1.24, *p* < 0.05), or ChABC alone plus ctr MO (Figures 2A vs. D, E; 17.8 ± 0.73 vs. 7.8 ± 1.24, *p* < 0.001). ChABC and RhoA MO individually had approximately equal effects on FLICA labeling. Once again, retrograde apoptotic signaling was inversely correlated with the regenerative probabilities of the individually identified RS neurons in all four treatment groups (Figures 2F, G; Ctr MO + Buffer: *r* = −0.7976, *p* < 0.0001; Ctr MO + ChABC: *r* = −0.9279, *p* < 0.0001; RhoA MO + Buffer: *r* = −0.8319, *p* < 0.001; RhoA MO + ChABC: *r* = −0.7930, *p* < 0.0001). The linear regression equation for Ctr MO treatment only was *Y* = −1.031 X + 90.81 (*p* < 0.0001); for Ctr MO plus ChABC treatment, it was *Y* = −1.280 X + 88.04 (*p* < 0.0001); for RhoA MO treatment only, it was *Y* = −0.9923 X + 74.27 (*p* < 0.0001); for RhoA MO plus ChABC treatment, it was *Y* = −0.7961 X + 53.62 (*p* < 0.0001) (Figure 2F).

According to the experiment design, at 4 weeks post-TX, the Ctr or RhoA MO treatment had been present for 4 weeks, while the treatment with ChABC in enzyme buffer or the enzyme buffer alone had been present for 3 weeks. Thus at 4 weeks post-TX, both RhoA MO and ChABC have similar potent antiapoptotic effects, but the combination of the two treatments has a greater effect than either alone, which suggests that CSPG signaling and RhoA have both overlapping and non-overlapping effects. We did not measure FLICA labeling at 10 weeks post-TX because by that time, some axotomized neurons may already be TUNEL positive or even dead (Shifman et al., 2008), and this might have led to spurious results.

### Effect of combinatorial treatments on initial axon retraction or regeneration *in vivo* at 2 weeks after spinal cord TX

**FIGURE 3:**
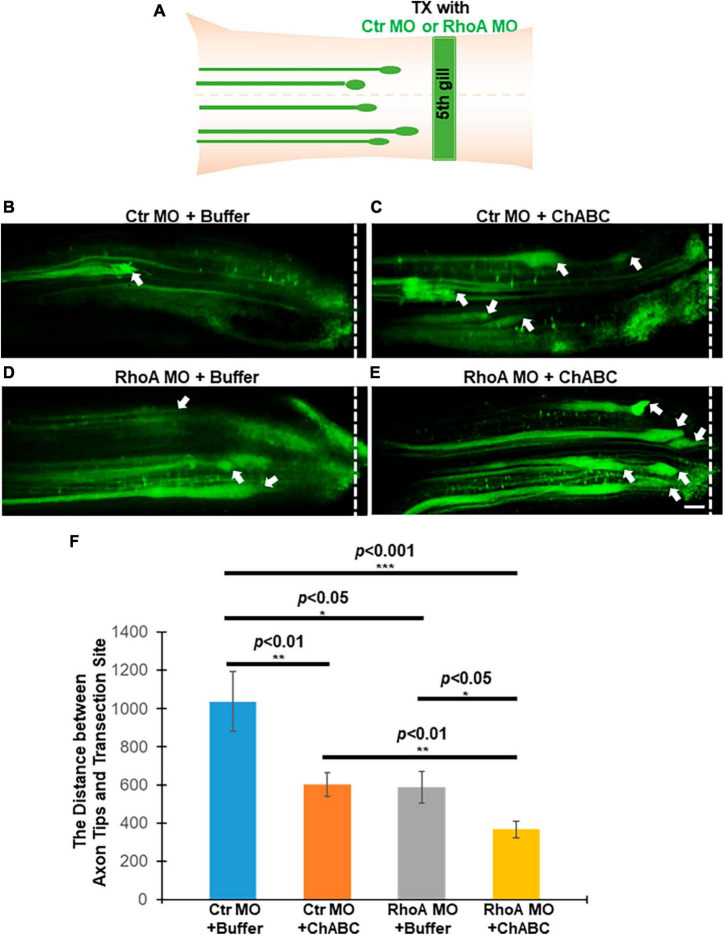
Effects of combinatorial treatments on axon retraction and/or regeneration *in vivo* after spinal cord TX at 2 weeks post-TX. Axon tips filled with MOs were shown under different combinatorial treatments. **(A)** Experimental design for detecting axon tips at 2 weeks after TX. **(B)** Ctr MO + buffer; **(C)** Ctr MO plus ChABC; **(D)** RhoA MO plus buffer; **(E)** RhoA MO plus ChABC. **(F)** The graph shows the distances between axon tips in the rostral stump and TX sites after different combinatorial treatment at 2 weeks post-TX. Smaller distances imply less retraction, greater regeneration, or both. *p < 0.05, **p < 0.01, and ***p < 0.001, *n* = 4 or 5 lampreys per group. Error bars: SEM. Scale bar: 100 μm. Arrows point to the axon tips, and the dotted lines show the TX site.

In mammals, after SCI, it is very difficult to track individual axon tips *in vivo*. Thus, the effects of RhoA and/or ChABC cannot be assessed in individual axons. We previously provided evidence for these effects *in vivo* by observing the behavior of individual neurons and axons in the lamprey CNS (Hu et al., 2017, 2021). We demonstrated that MO-mediated knockdown of RhoA *in vivo* reduced axon retraction at 1 and 2 weeks post-TX by approximately 50%, and that at 2 weeks post-TX, the average distance between the axon tips and the TX site was greater than it had been at the end of 1 week; i.e., at 1 week, axotomized axons are still retracting (Hu et al., 2017). Thus, either RhoA MO knockdown reduced the rate of retraction of the injured axon tips, or accelerated axon regeneration beginning at some point between 1 and 2 weeks post-TX. ChABC treatment also had beneficial effects on the injured axons, reducing the distance between the axon tips and TX sites at 2 weeks post-TX (Hu et al., 2021). In the present study, application of fluorescently labeled MOs, either the Ctr or RhoA MO, at the time of injury labels the injured axon tips. Thus we could measure the distances between axon tips and TX sites (Figure 3A), and found that treatment with either Ctr MO plus ChABC, or RhoA MO plus enzyme buffer, or RhoA MO plus ChABC, each resulted in reducing the distances between the axon tips and the TX sites, compared with Ctr MO plus enzyme buffer alone (Figures 3B vs. C, 1037 ± 155.3 μm vs. 601.6 ± 64.1 μm, *p* < 0.01; Figures 3B vs. D, 1037 ± 155.3 μm vs. 588.8 ± 84.3 μm, *p* < 0.05; Figures 3B vs. E, 1037 ± 155.3 μm vs. 366.7 ± 44.1 μm, *p* < 0.001; Figure 3F). ChABC (plus ctr MO) and RhoA MO (plus enzyme buffer) individually, had comparable effects (Figures 3C vs. D, 601.6 ± 64.1 μm vs. 588.8 ± 84.3 μm; Figure 3F), but combined, the distance in axon retraction was reduced compared with either treatment alone (Figures 3C vs. E, 601.6 ± 64.1 μm vs. 366.7 ± 44.1 μm, *p* < 0.01; Figures 3D vs. E, 588.8 ± 84.3 μm vs. 366.7 ± 44.1 μm, *p* < 0.05; Figure 3F). These findings suggest that the combination of RhoA knockdown and ChABC have both overlapping and non-overlapping effects in reducing the rate of retraction of the injured axon tips or accelerating their regeneration in the early after transection, while the axon tips of the large identified RS neurons are still in the proximal (rostral) stump of spinal cord.

### Effect of combinatorial treatments on axon regeneration beyond the TX site for identified RS neurons

**FIGURE 4:**
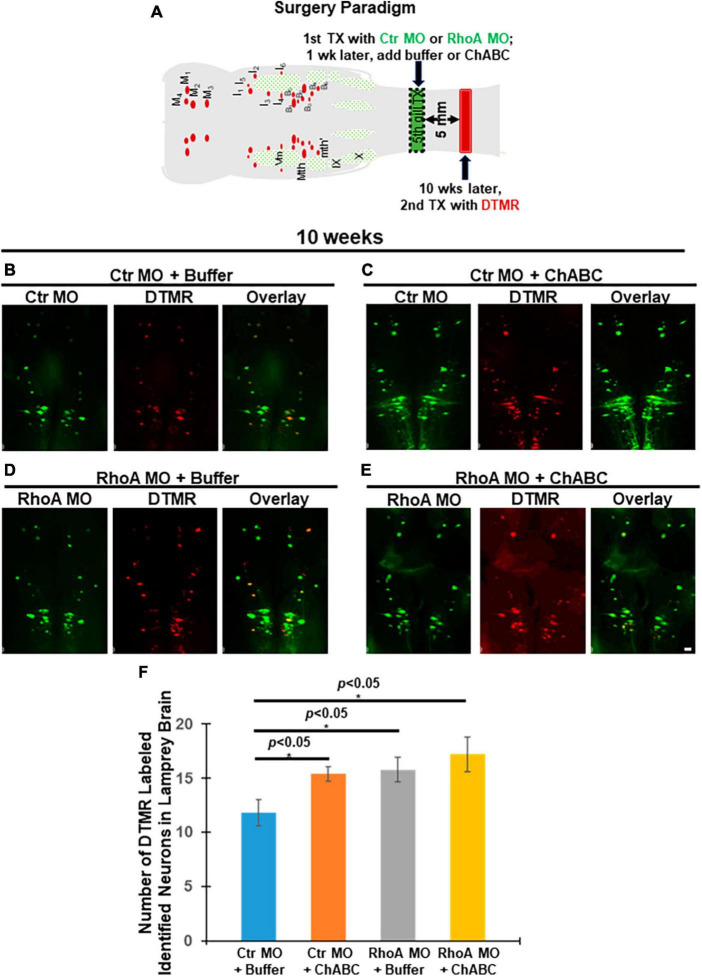
Effects of combinatorial treatments on axon regeneration past the TX site *in vivo* at 10 weeks post-TX. Regenerated RS neurons in the brainstem were retrogradely labeled by DTMR (red) from 5 mm caudal to the initial injury at 10 weeks post-TX. **(A)** Experimental design for detecting neurons whose axons have regenerated. Ten weeks after a complete spinal cord TX, DTMR was applied to a second TX 5 mm caudal to the first, and 5 more days allowed for retrograde transport of the dye. **(B)** Ctr MO plus buffer. **(C)** Ctr MO plus ChABC. **(D)** RhoA MO plus buffer. **(E)** RhoA MO plus ChABC. **(F)** Graph showing an increased number of retrogradely labeled neurons in brainstems of animals treated with Ctr MO plus ChABC, RhoA MO plus buffer, RhoA MO plus ChABC, compared to Ctr MO plus buffer. *p < 0.05, *n* = 5 lampreys per group. Error bars: SEM. Scale bar: 100 μm.

At 10 weeks post-TX, a retrograde tracer DTMR (Red) was introduced from a second transection site 5 mm caudal to the first lesion (Figure 4A) to retrogradely label RS neurons in the brainstem whose axons had regenerated. Groups of animals receiving Ctr MO plus ChABC, RhoA MO plus enzyme buffer and RhoA MO plus ChABC all showed increases in the number of DTMR-labeled identified RS neurons, compared with the control group treated with Ctr MO plus enzyme buffer (Figures 4B vs. C, 11.8 ± 1.24 vs. 15.4 ± 0.68; Figures 4B vs. D, 11.8 ± 1.24 vs. 15.8 ± 1.16; Figures 4B vs. E, 11.8 ± 1.24 vs. 17.2 ± 1.63; *p* < 0.05; Figure 4F). ChABC treatment alone (i.e., plus ctrl MO) and RhoA MO alone (i.e., plus enzyme buffer), had approximately equal effects (Figures 4C vs. D, 15.4 ± 0.68 vs. 15.8 ± 1.16; Figure 4F). The combination of RhoA MO and ChABC showed the greatest effect, but the increases over each treatment individually did not reach statistical significance (Figures 4C, D vs. E, 15.4 ± 0.68 and 15.8 ± 1.16 vs. 17.2 ± 1.63; Figure 4F).

### Akt-T308 phosphorylation changes in individual RS neurons after combinatorial treatments

**FIGURE 5:**
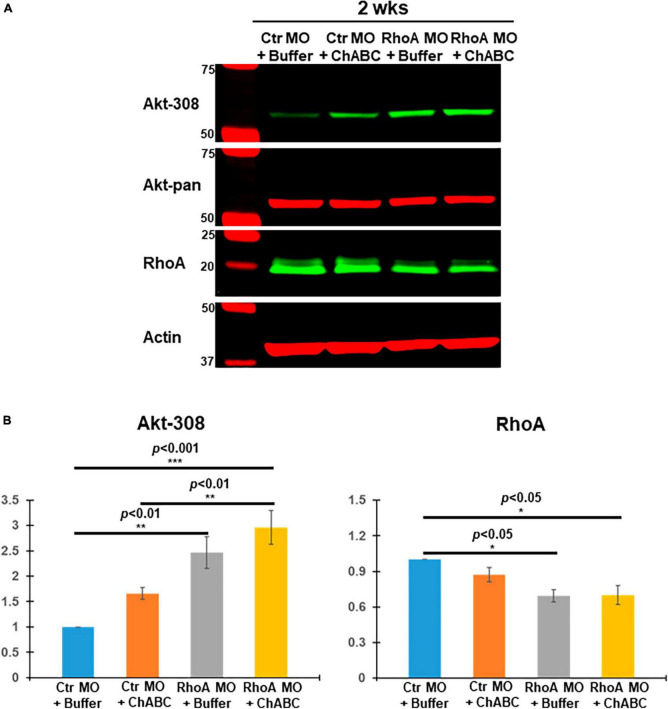
Akt and RhoA changes *in vivo* after different combinatorial treatments. **(A)** Brain homogenates from 2 weeks post-TX, treated with Ctr MO plus buffer, Ctr MO plus ChABC, RhoA MO plus buffer or RhoA MO plus ChABC were examined by Western blots and probed with mAbs against pAkt-308, total Akt (Akt-pan) or RhoA, and actin as a loading control. **(B)** Graph showing that RhoA MO plus buffer and RhoA MO plus ChABC treatment each produced a significant increase in pAkt-308 and a significant decrease in RhoA expression compared to the CtrMO plus buffer. Although ChABC also was associated with increased pAkt-308 immunoactivity, this did not reach statistical significance, either by itself or in addition to RhoA-MO. *p < 0.05, **p < 0.01, ***p < 0.001, *n* = 4 lampreys per group. Error bars: SEM.

Akt is an important molecular target signal downstream of CSPG receptors in neurons *in vitro* (Fisher et al., 2011), and its activation (phosphorylation) promotes axon regeneration after SCI *in vivo*. We have reported that digestion of CSPGs with ChABC after SCI increased Akt phosphorylation levels at threonine 308 (pAkt-308) in individually identified RS neurons of lamprey brains post-TX (Hu et al., 2021). When we added supplementary CSPGs onto the TX site after SCI, Akt phosphorylation at threonine 308 was reduced, compared to TX only or to TX plus ChABC (Supplementary Figure 3). RhoA knockdown also enhanced Akt phosphorylation at threonine 308 (Supplementary Figure 4). Thus, pAkt-308 is a critical molecule under the modulation of CSPGs and CSPG receptors, while RhoA also contributes to the phosphorylation of Akt *in vivo*. Therefore, in the current study, the effects of different combinatorial therapies on Akt were evaluated. At 2 weeks post-TX, Ctr MO plus ChABC, RhoA MO plus enzyme buffer and RhoA MO plus ChABC all increased Akt phosphorylation at threonine 308 (Figure 5). RhoA MO plus ChABC had the strongest effect (Figure 5). This treatment-induced activation of Akt is consistent with its reduction of axon retraction or enhancement of axon regeneration described above (Figure 3). Correspondingly, we found a significant reduction in RhoA expression in the RhoA MO plus Buffer and RhoA MO plus ChABC groups, which is consistent with our previous report on RhoA knockdown. However, ChABC alone (i.e., plus only ctr MO) neither further increased Akt activation nor reduced RhoA expression levels at 2 weeks post-TX, probably due to the relatively brief period of ChABC application (1 week).

**FIGURE 6:**
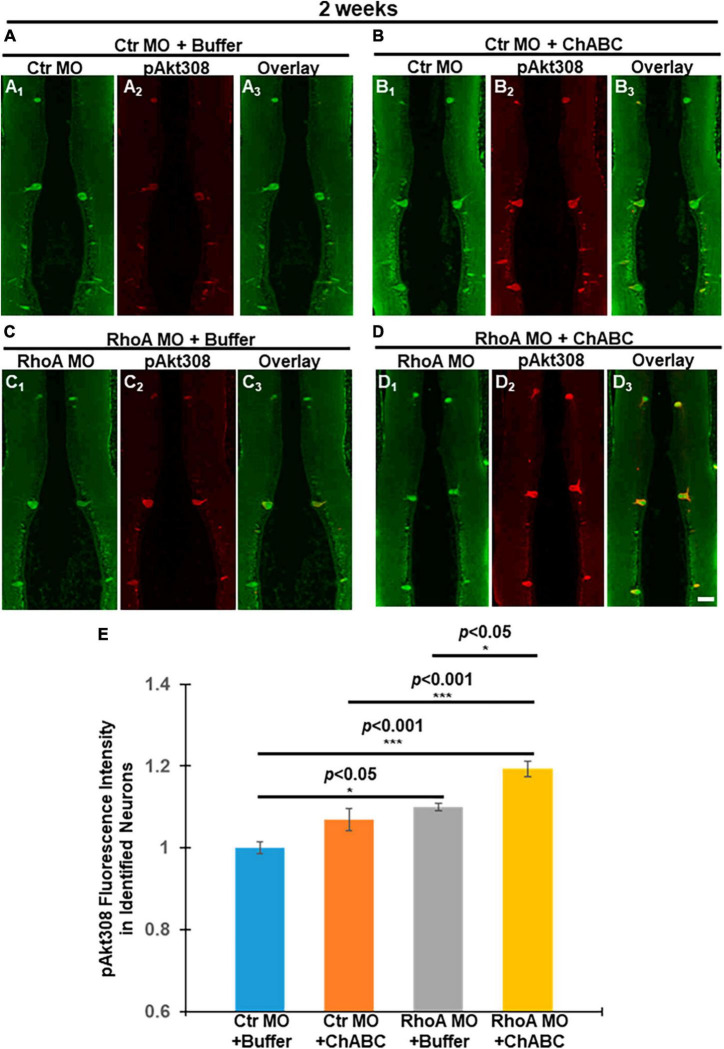
Akt phosphorylation changes in individual RS neurons after different combinatorial treatments at 2 weeks post-TX. (1 week ChABC, 2 weeks MO) Akt phosphorylation at Threonine 308 (T308) was assessed by immunofluorescence staining. Paraffin sections of lamprey brains were used after spinal cord TX and different combinatorial treatments for 2 weeks. Individual identified RS neurons were labeled with either Ctr MO or RhoA MO (Green). pAkt-308 expressions are shown in Red. **(A_1_–A_3_)**, Ctr MO plus buffer. **(A_1_)** Ctr MO fluorescence. **(A_2_)** pAkt-308 staining. **(A_3_)** Overlay of Ctr MO and pAkt-308 immuno-fluorescence. **(B_1_–B_3_)**, Ctr MO plus ChABC. **(B_1_)**, Ctr MO. **(B_2_)** pAkt-308. **(B_3_)** Overlay of Ctr MO and pAkt-308 fluorescence. **(C_1_–C_3_)**, RhoA MO plus buffer. **(C_1_)** RhoA MO **(C_2_)** pAkt-308. **(C_3_)** Overlay of RhoA MO and pAkt-308 immuno-fluorescence. **(D_1_–D_3_)**, **(D)** RhoA MO plus ChABC. **(D_1_)** RhoA MO, **(D_2_)** pAkt-308. **(D_3_)**. **(D_3_)** Overlay of RhoA MO and pAkt-308 immuno-fluorescence. We used MO fluorescence to label each identified RS neuron in order to quantify the pAkt-308 fluorescence intensity. **(E)** Graph shows quantitative fluorescence intensity of pAkt-308 in identified RS neurons in lamprey brains after different combinatorial treatments. *p < 0.05, ***p < 0.001, *n* = 9∼14 lampreys per group. Error bars: SEM. Scale bar: 100 μm.

Since Western blots cannot specify whether Akt activation occurs specifically in axotomized neurons, we next investigated Akt activation status in individually identified RS neurons by quantitative immunofluorescence. At 2 weeks post-TX and application of fluorescently tagged Ctr MO or RhoA MO retrogradely labeled RS neurons, whereas their nearby glial cells and small locally projecting neurons lacking long spinal cord projections were not labeled. One week later, the TX site was treated with either control enzyme buffer or ChABC. Lampreys were sacrificed at 2 weeks post-TX, and their brains fixed and processed for paraffin sectioning. The expression levels of pAkt-308 in individual RS neurons were quantified by immunofluorescence. Individual identified RS neurons were recognized with the retrogradely labeled Ctr MO or RhoA MO (Green, Figures 6A–D), and then by pAkt-308 immunofluorescence (Red, Figures 6A1–D1). Akt phosphorylation intensity was quantified (Figures 6A2–D2). Treatment with RhoA MO plus ChABC showed the most robust Akt phosphorylation increase in these neurons. Each of the four treatment groups showed an increase in Akt activation, although the increase associated with ChABC alone over ctr medium did not reach statistical significance. RhoA MO and ChABC had similar efficacies. The combination of RhoA MO and ChABC was significantly more effective than either treatment alone. These findings suggest that RhoA MO knockdown has additive effects over ChABC treatment on the activation of Akt and that RhoA might act upstream of Akt. Moreover, the patterns of Akt phosphorylation in identified RS neurons after different treatments were similar to the patterns of retrograde apoptosis signaling changes at 2 and 4 weeks post-TX (Figures 1E, 2E vs. 6E), which indicates that Akt activation might reduce the retrograde apoptosis signaling and thereby contribute to neuronal survival.

## Discussion

We have investigated whether ChABC adds to the neuronal protective effects of RhoA knockdown *in vivo*. Four different treatment combinations were applied in lampreys for 2, 4, and 10 weeks after SCI: control MO (plus enzyme buffer), control MO plus ChABC (in enzyme buffer), RhoA MO (plus enzyme buffer), and RhoA MO plus ChABC (in enzyme buffer). In all cases, the therapeutic effects of either RhoA MO or ChABC on apoptotic signaling and axon regeneration were better than the effects of their respective controls, and the combination of RhoA MO plus ChABC showed more beneficial effects than either treatment alone. At 2 weeks post-TX, the effects of ChABC on apoptotic signaling were not statistically significant; nor did the addition of ChABC to RhoA MO significantly increase the therapeutic effect (Figure 1), although ChABC did reduce axon retraction or enhance proximal axon regeneration. This is not surprising because in our previous study in lamprey, ChABC treatment *in vivo* required 2 weeks to reduce retrograde apoptotic signaling (Hu et al., 2021), whereas in the current experimental design, at 2 weeks post-TX, the Ctr or RhoA MO treatment had been present for 2 weeks, while the enzyme buffer or ChABC dissolved in enzyme buffer had been present for only 1 week. By 4 weeks, post-TX, the therapeutic effects of ChABC on both apoptotic signaling and axon retraction (or regeneration in the proximal stump) were statistically significant, and RhoA MO plus ChABC was significantly better than either alone (Figure 3).

Although at 10 weeks post-TX, either RhoA MO or ChABC enhanced axon regeneration, as judged by retrograde labeling from caudal to the original TX, and the combination of RhoA MO plus ChABC had a greater beneficial effect than either treatment alone, the effect of combining the treatments did not reach statistical significance (Figure 4). Perhaps the RhoA MO and ChABC combination might not have much additive long-term effect on axon regeneration, but, there might be other explanations. For example, the method of measuring axon regeneration by retrograde transport of tracer may underestimate the total amount of axon regeneration because it does not detect identified RS neurons whose axons regenerated less than 5 mm beyond the first TX. Nor does it distinguish between axons that had regenerated only as far as the site of retrograde tracer application (5 mm caudal to the original lesion) and axons that had regenerated much further than that. Regeneration much longer than 5 mm has been reported at long recovery times in large RS axons of lamprey (Davis and McClellan, 1994). These technical limitations might create a ceiling effect for measurement of axon regeneration with the method used here. If in addition to the axons of identified RS neurons, we had also assessed regeneration of smaller axons, by anterograde (Zhang et al., 2020) and/or retrograde tracing (Sobrido-Cameán et al., 2020), the number of tested axons would have been much greater and the difference between the RhoA MO plus ChABC combined treatment and either treatment alone might have reached statistical significance.

RhoA MO + ChABC treatment enhanced the pAkt-308 phosphorylation greatly, compared to all the other three treatment groups, not only in brain tissue homogenates (Figure 5) but also in individually identified RS neurons (Figure 6). The additive effect on Akt phosphorylation matched the beneficial effects on neuronal survival and axon regeneration.

### Current research on combinatorial treatments in SCI

In the past decade, many combinations of ChABC with other therapeutic tools have been tried, such as peripheral nerve grafts, cell transplants, growth factors (NGF, GDNF, NT3), low level laser treatment and rehabilitation (Muir et al., 2019). The effects on axon regeneration after SCI were synergistic with the actions of ChABC. Here we will focus on combinations of ChABC with pharmacological and molecular treatments analogous to RhoA knockdown, but will not discuss peripheral nerve grafts or cell transplants.

Implanted microspheres releasing a combination of ChABC, GDNF, and anti-Nogo A antibodies improved locomotor scores in SCI rats (Zhang et al., 2013). Cortical somatosensory evoked potentials also were augmented, but histological changes were not reported. In a T10 contusion model of partial SCI in rat, ChABC combined with adeno-associated virally released neurotrophin 3 (AAV-NT3) treatment increased the strength of detour synaptic inputs from corticospinal axons onto dorsomedial interneurons that connect to motor neurons caudal to the lesion. The enhanced intracellularly recorded synaptic inputs onto motor neurons were accompanied by increased immunoreactivity of 5-HT-positive fibers in lumbar dorsal and ventral horns and improved locomotor function (Hunanyan et al., 2013).

Antisense cDNA for vimentin, a major component of the cytoskeleton of reactive glia, was used to knock down vimentin expression. When this was combined with ChABC, it reduced glial scar and cystic cavity formation after SCI in rats (Xia et al., 2008). In a follow-up report, the investigators found that this combined treatment promoted axon regeneration and functional recovery after SCI, indicating that axon regeneration may be promoted by modified physical and biochemical characteristics of intra- and extracellular architecture in glial scar tissues (Xia et al., 2015).

When 660 nm low-level laser radiation (LLLT), an inflammation-inhibiting treatment, was combined with ChABC, this had a greater effect in reducing cavity size, improving myelination, and increasing the number of axons around the cavity, than either treatment alone. The same was true regarding the effects of the combination treatment compared with LLLT or ChABC alone in reducing the expression of GSK3β, CSPG and AQP4, as well as in improving functional recovery (Janzadeh et al., 2017).

Thus, in all these studies, the beneficial effects of ChABC combined with other treatments were greater than those of individual therapies. Our study also showed that the ChABC plus RhoA knockdown had better effects on axon regeneration and neuronal survival after SCI than either therapy alone.

### Combinatorial therapy reduces retrograde apoptotic signaling

The poly-caspase FLICA we used can detect caspase 1, 3, 4, 5, 6, 7, 8, and 9. Previously, we found that digestion of CSPGs with ChABC significantly reduced the number of polycaspases positive RS neurons at 2 weeks post-TX and the number of those caspase 3 positive RS neurons at 4 weeks post-TX (Hu et al., 2021). Based on these findings, we concluded that digestion of CSPGs with ChABC reduces retrograde apoptotic signaling. At 1 week after optic nerve injury, the death of axotomized RGCs can be completely stopped by intraocular injection of C3 (inactivating RhoA) (Bertrand et al., 2005), indicating that RhoA could mediate short-term retrograde neuronal death. Previously, we showed that knockdown of RhoA with MO *in vivo* significantly reduced retrograde apoptotic signaling as indicated by FLICA labeling in identified RS neurons after SCI in lamprey (Hu et al., 2017). The reduction was observed at 2 and 4 weeks post-TX, suggesting that RhoA plays a critical role in delayed retrograde neuronal death after SCI. Consistently, in the present study, the number of identified RS neurons containing activated polycaspases decreased significantly by RhoA MO + ChABC at 4 weeks post-TX, compared to all the other treatment combinations. Our study indicates that RhoA knockdown increases Akt phosphorylation levels in our system (Supplementary Figure 4). Further elucidation of the signaling pathway responsible for this cell death could help us to understand how to protect neurons from axotomy-induced neuronal death.

### Combinatorial therapy promotes axon regeneration

At 2 weeks post-TX, Ctr MO plus ChABC reduced the distances between the axon tips and TX sites (Figures 3A–C, F). RhoA MO plus buffer had similar effects (Figures 3A–D, F). These data are consistent with what we have published previously on the effects of ChABC and RhoA MO treatments individually (Hu et al., 2017, 2021). At the same time, the RhoA MO plus ChABC combinatorial therapy worked better than either treatment alone, reducing the distance between the axon tips and the TX site by more than 60%, compared with controls, whereas individually, the effects were less than 40% (Figures 3E, F). This suggests that at least some of the early inhibitory effects of CSPGs on regeneration (or in promoting axon retraction) are independent of RhoA signaling. Longer-term axon regeneration of RS axons beyond the TX site was evaluated by retrograde labeling of RS neurons at 10 weeks post-TX. Although the effect of RhoA MO plus ChABC was greater than that of either treatment alone (∼45 vs. ∼33% increase in the number of retrogradely labeled neurons, compared with controls), the difference did not reach statistical significance. Perhaps there are some ceiling effects over long-term axon regeneration after SCI, which could cause a requirement for larger sample sizes to detect significant differences. It also is possible that limitations of the retrograde labeling method make detection of differences less accurate. The method only detects axon regeneration longer than 5 mm, whereas many axons regenerate shorter distances (Rovainen, 1976; Selzer, 1978; Yin and Selzer, 1983, 1984), and would be missed.

The studies on mammalian models of SCI that we have summarized above suggest that combining ChABC with different manipulations enhanced axon sprouting and functional recovery after SCI. But due to the complexity of the mammalian CNS, and the use of partial injury models, it is unclear whether the additive beneficial effect was due entirely to collateral sprouting by spared axons, or also involves true regeneration of injured axons. In the current study on lampreys, we performed complete spinal cord TXs, and labeled only transected axons and their parent cell bodies. At an early stage (2 weeks) after TX, the axon tips were filled with MO fluorescence, so their locations could be assessed directly. The results indicated that early after TX, RhoA knockdown or CSPG digestion either reduced axon retraction or enhanced axon regeneration, or both, and that the effects of the combined treatment were better than either one alone. The long-term axon regeneration quantification is much stricter since we used 5 mm axon regeneration as a cut-off, thereby the increased growth of RS axons beyond the lesion was due to true regeneration. Thus, our study further highlighted the critical role of ChABC combining with other manipulations (specifically RhoA knockdown) in the treatment of SCI.

### Combinatorial therapy affects intracellular signaling molecules

In neuronal cultures, CSPG reduces the Akt phosphorylation (Fisher et al., 2011; Silver et al., 2014; Ohtake et al., 2016). CSPGs application enhanced 4E-BP1 that is a negative signal along the Akt pathway (Walker et al., 2012). Akt activation can promote optic nerve axon regeneration and survival of retinal ganglion cells (RGC) (Yang et al., 2014). Akt activation enhanced axon regeneration in the sensory neurons in live Drosophila larvae (Wang et al., 2020). Previously, we reported that ChABC treatment greatly promoted axonal regeneration and protected neurons from undergoing retrograde apoptosis after SCI, which was accompanied by widespread enhancement of Akt activation (pAkt-308) in the brain (Hu et al., 2021). The increase in Akt phosphorylation was confirmed in individual identified RS neurons (Hu et al., 2021). In the present report, we found that CSPGs application *in vivo* inhibited Akt phosphorylation while ChABC application enhanced it after SCI (Supplementary Figure 3), which confirmed that Akt acts under CSPGs and CSPG receptors. We also found that RhoA MO knockdown increased the Akt activation (Supplementary Figure 4), indicating that RhoA plays a role over the status of Akt *in vivo*. Consistently, the RhoA MO + ChABC treatment has the greatest Akt phosphorylation among all the different combination treatments (Figure 6). It is well known that RhoA is critical in mediating the inhibitory effect of axon growth by CSPGs (Fournier et al., 2003; Fu et al., 2007; Dill et al., 2008). The additive effects of RhoA MO + ChABC treatment also support this role of RhoA in axon growth.

The present findings on the effects of combinatorial RhoA plus ChABC treatments on retrograde apoptotic signaling, axon retraction, axon regeneration and intracellular signaling molecules shed light on the mechanisms by which neurons respond to axotomy after SCI, and may suggest future clinical applications.

## Data availability statement

The original contributions presented in the study are included in the article/Supplementary material, further inquiries can be directed to the corresponding author.

## Ethics statement

The animal study was approved by the Temple University Institutional Animal Care and Use Committee. This study was conducted in accordance with the local legislation and institutional requirements.

## Author contributions

JH: Conceptualization, Data curation, Formal analysis, Investigation, Methodology, Software, Visualization, Writing – original draft. GZ: Methodology, Validation, Writing – review and editing. WR: Methodology, Validation, Writing – review and editing. L-QJ: Methodology, Validation, Writing – review and editing. MS: Funding acquisition, Project administration, Resources, Supervision, Writing – review and editing, Conceptualization, Visualization.

## Supplementary material

Supplementary Figure 1 ChABC treatment of lamprey spinal cord after TX. Stumps of digested CSPGs were labeled with mAb 2B6 at different times after spinal cord TX and imaged in sagittal sections of spinal cord by colorimetric immunohistochemistry. **(A)** Sagittal section of a spinal cord that was transected and treated with ChABC for 1 day. **(B)** Sagittal section of a spinal cord that was transected and treated with ChABC for 1 week. **(C)** Sagittal section of a spinal cord that was transected and treated with ChABC for 2 weeks. Scale bar: 1 mm.

Supplementary Figure 2 RhoA knockdown efficiency. IHC show neurofilament **(A,B)** and RhoA **(C,D)** expressions in transversely sectioned spinal cords after Ctr MO **(A,C)** and RhoA MO **(B,D)** application for 2 weeks. **(A,C)** are adjacent transverse sections (10 μm per section) from the same spinal cord. **(B,D)** Also are adjacent sections.

Supplementary Figure 3 Modulation of Akt activity by CSPGs. Western blots show that ChABC digestion enhanced Akt phosphorylation at T308 after SCI at 1 and 2 weeks post-TX, while addition of CSPGs over the TX site inhibited Akt phosphorylation.

Supplementary Figure 4 Changes in RhoA expression and Akt activation in lamprey brain and spinal cord after morpholino treatment. RhoA MOs reduced RhoA expression levels in brain and spinal cord by 2 weeks post-TX, but did not affect the expression of total Akt (Akt-pan). Interestingly, Akt-T308 phosphorylation was increased, indicating that RhoA knockdown activates Akt. Actin was used as a loading control.
