# Supplementary figures and images for: Combined RhoA morpholino and ChABC treatment protects identified lamprey neurons from retrograde apoptosis after spinal cord injury
Source: Front Cell Neurosci. 2023 Dec 21;17:1292012. doi: 10.3389/fncel.2023.1292012 (PMC10764559; doi:10.3389/fncel.2023.1292012)

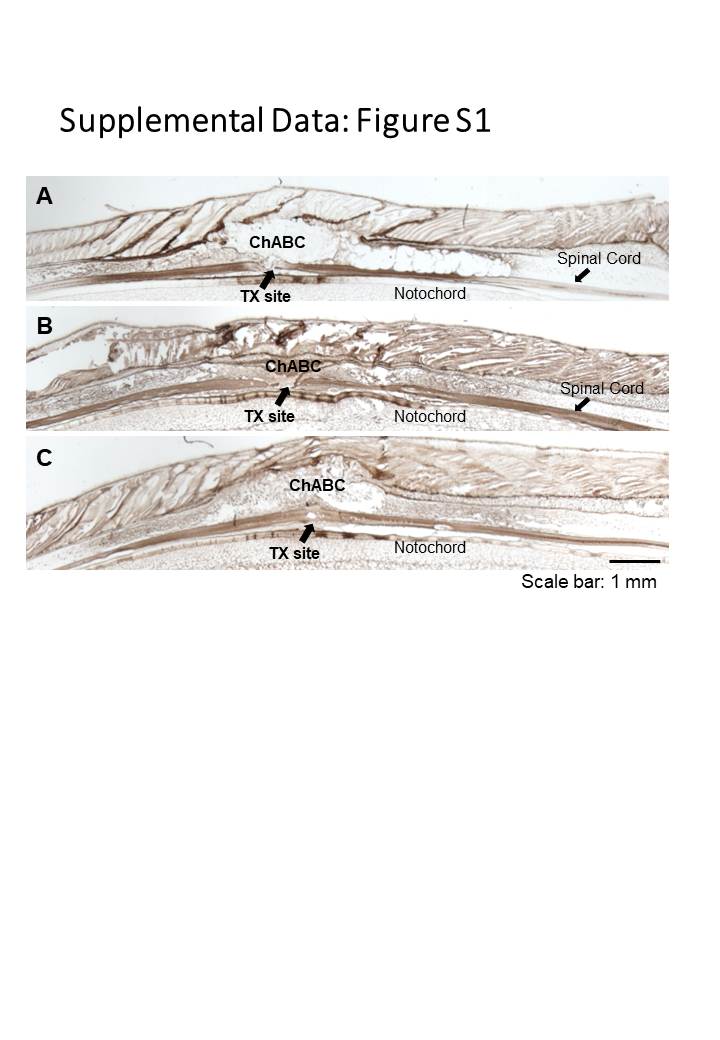

Supplement: Supplementary Figure 1 — ChABC treatment of lamprey spinal cord after TX. Stumps of digested CSPGs were labeled with mAb 2B6 at different times after spinal cord TX and imaged in sagittal sections of spinal cord by colorimetric immunohistochemistry. (A) Sagittal section of a spinal cord that was transected and treated with ChABC for 1 day. (B) Sagittal section of a spinal cord that was transected and treated with ChABC for 1 week. (C) Sagittal section of a spinal cord that was transected and treated with ChABC for 2 weeks. Scale bar: 1 mm. [file Image_1.TIF]

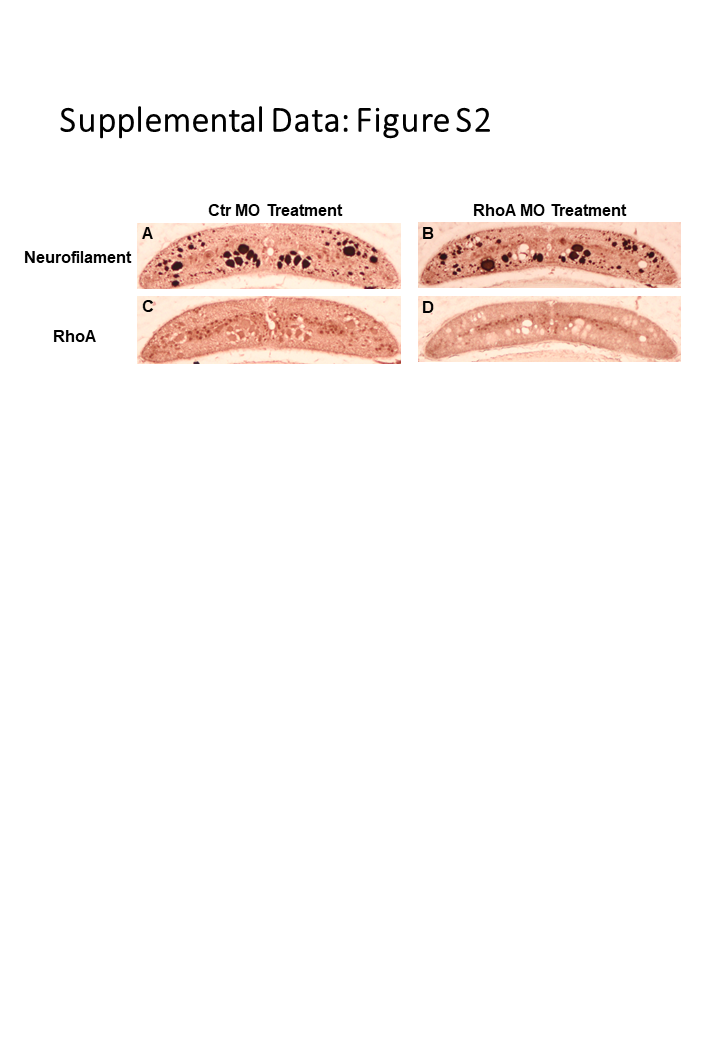

Supplement: Supplementary Figure 2 — RhoA knockdown efficiency. IHC show neurofilament (A,B) and RhoA (C,D) expressions in transversely sectioned spinal cords after Ctr MO (A,C) and RhoA MO (B,D) application for 2 weeks. (A,C) are adjacent transverse sections (10 μm per section) from the same spinal cord. (B,D) Also are adjacent sections. [file Image_2.TIF]

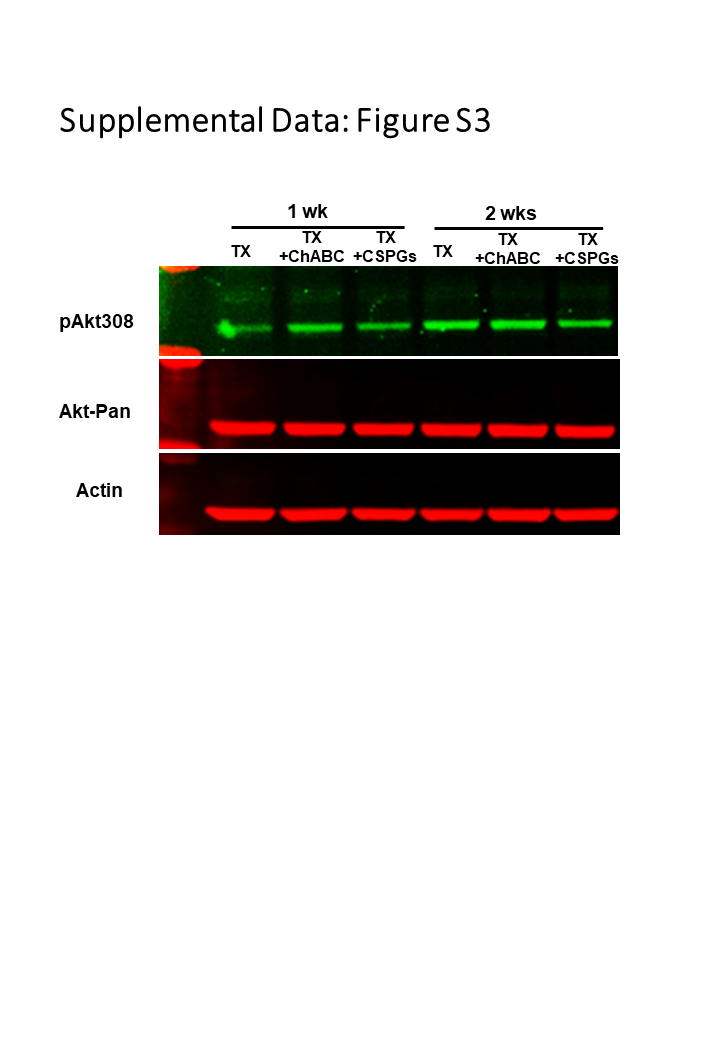

Supplement: Supplementary Figure 3 — Modulation of Akt activity by CSPGs. Western blots show that ChABC digestion enhanced Akt phosphorylation at T308 after SCI at 1 and 2 weeks post-TX, while addition of CSPGs over the TX site inhibited Akt phosphorylation. [file Image_3.TIF]

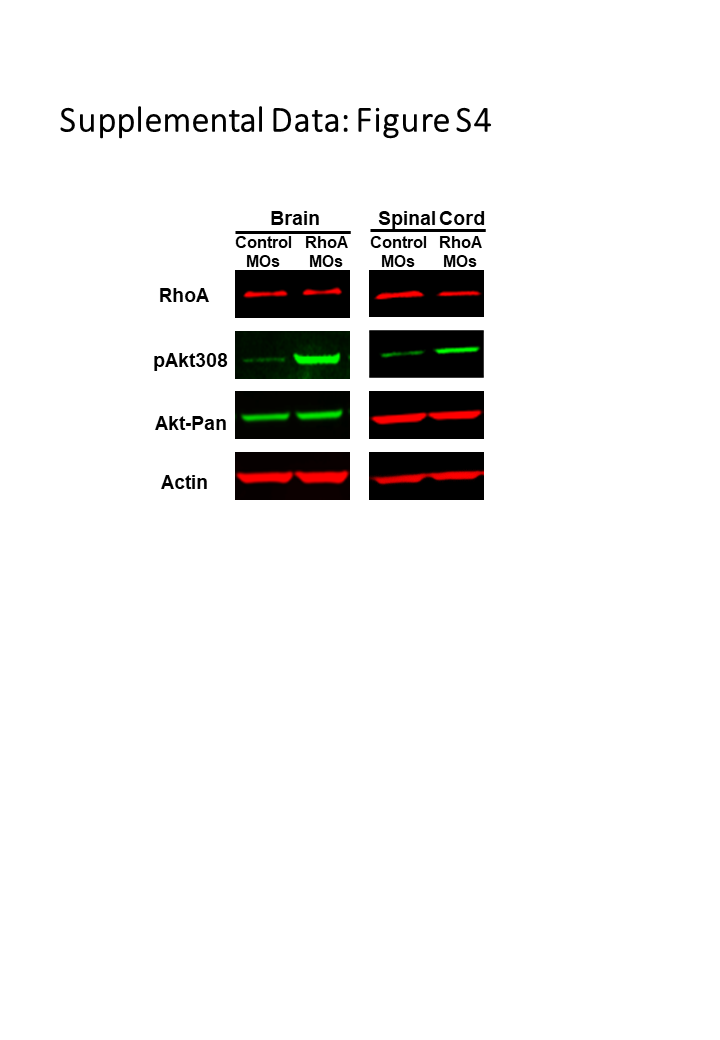

Supplement: Supplementary Figure 4 — Changes in RhoA expression and Akt activation in lamprey brain and spinal cord after morpholino treatment. RhoA MOs reduced RhoA expression levels in brain and spinal cord by 2 weeks post-TX, but did not affect the expression of total Akt (Akt-pan). Interestingly, Akt-T308 phosphorylation was increased, indicating that RhoA knockdown activates Akt. Actin was used as a loading control. [file Image_4.TIF]
